# Effects of Fibroblast Growth Factor 23 (FGF23) on the Cardiovascular System: A Review of Literature

**DOI:** 10.7759/cureus.97147

**Published:** 2025-11-18

**Authors:** Diego Ivan Diaz-Haaz, Eleasib Alejandro Espinoza-Pérez, Jesús Armando Aguilar-Alonso, Mauricio Megchún-Hernández, Fernando Gonzalez-Diaz, Néstor Rodolfo García-Chong

**Affiliations:** 1 Faculty of Human Medicine "Dr. Manuel Velasco Suárez" Campus II, Universidad Autónoma de Chiapas, Tuxtla Gutiérrez, MEX; 2 Department of Nephrology, Instituto Mexicano del Seguro Social, Hospital General de Zona No. 2, Tuxtla Gutiérrez, MEX; 3 Pediatrics, Hospital de Especialidades Pediátricas, Tuxtla Gutiérrez, MEX; 4 Faculty of Medicine, Benemérita Universidad Autónoma de Puebla, Puebla, MEX

**Keywords:** cardiovascular disease, chronic kidney disease, fgf23, hypertension, left ventricular hypertrophy, myocardial infarction, phosphate

## Abstract

Fibroblast growth factor 23 (FGF23) is a hormone that plays a crucial role in phosphate metabolism; its synthesis increases with phosphate intake. The effect of FGF23 is reduced by the decrease in Klotho protein in patients with chronic kidney disease (CKD). As a result, there is less phosphate excretion and, consequently, an increase in serum FGF23 levels. Several studies have shown that elevated FGF23 levels are associated with an increased risk of cardiovascular events. This is a consequence of the various alterations it causes at this level, including arterial stiffness, increased pulse wave velocity, left ventricular hypertrophy, cardiac tissue fibrosis, atrial fibrillation, and atherosclerosis, resulting in increased cardiovascular mortality and all-cause mortality. The pathophysiological mechanisms by which FGF23 generates all these alterations are novel and will be discussed in this review. Therapeutic strategies to reduce FGF23 levels include low-phosphate diets, some intestinal phosphate binders, calcimimetics, dialysis therapies, and some other medications that require further research to evaluate their effectiveness.

## Introduction and background

FGF23 belongs to a family of growth factors involved in various functions (cell proliferation, migration, differentiation, mitogenesis, angiogenesis, embryogenesis, among others). Of the 22 members, FGF23 is the most studied [1]. It is a protein of 251 amino acids, has a total length of 32 kDa, and is produced by osteocytes and osteoblasts under physiological conditions. It acts on various tissues but has a predominant function in the kidneys and parathyroid glands. It performs its function by binding to fibroblast growth factor 23 (FGFR) receptors, of which four types have been described. The first three of which have two isoforms (b and c), and type 4 has only one isoform. FGF23 binds to the FGFR1c and FGFR3c receptors, requiring the α-Klotho (hereafter, referred to as Klotho) protein as a co-receptor, whereas its binding to the FGFR4 receptor does not require Klotho [2, 3]. FGF23 consists of an N-terminal domain and a C-terminal domain, where Klotho interacts as a cofactor and plays a fundamental role in maintaining phosphate homeostasis, given that its effects include increasing its excretion by the kidney and decreasing its intestinal reabsorption. Multiple studies have demonstrated an association between elevated FGF23 concentrations and increased cardiovascular mortality [4,5]. Several diseases have been described that present with elevated levels of FGF23, such as X-linked hypophosphatemia (XLH), autosomal dominant and recessive hypophosphatemic rickets, fibrous dysplasia, tumor-induced osteomalacia, and chronic kidney disease (CKD). It is in the latter that the most deleterious effects of FGF23 on the cardiovascular system have been observed.

## Review

FGF23 stimulants

**Figure 1:**
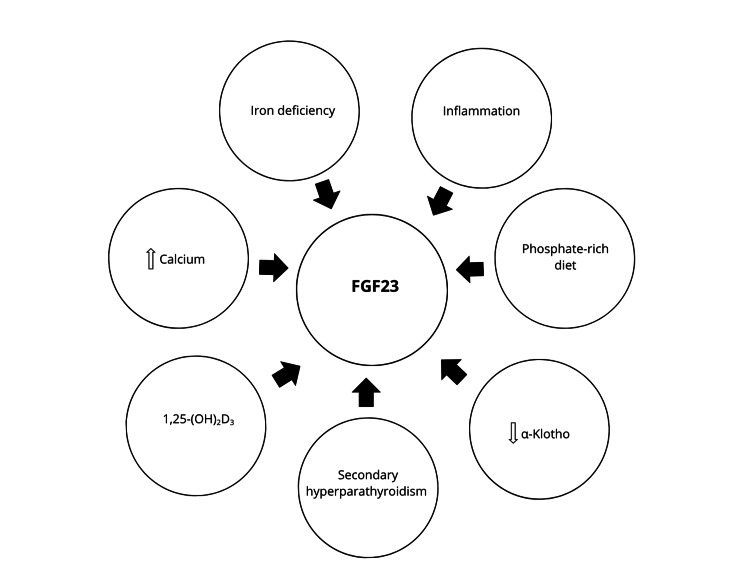
Stimuli and regulatory factors of fibroblast growth factor 23 (FGF23) production. Image created by the authors.

Dietary phosphate intake stimulates the production and secretion of FGF23 by osteocytes, which reduces phosphate reabsorption by decreasing the expression of sodium/phosphate cotransporters (NaPi-2a and NaPi-2c) in the proximal tubules of the kidney [6]. Consuming a calcium-enriched diet contributes to an increase in FGF23. Calcitriol increases the expression of the nuclear receptor associated with type 1 protein (Nurr1) in bone cells, which in turn promotes the production of FGF23 [7]. The decline in Klotho expression observed in CKD reduces the ability to activate its receptor in the kidneys, which causes lower phosphate excretion and, therefore, an increase in serum phosphate levels. Increased parathyroid hormone (PTH) in CKD stimulates FGF23 secretion through the Nurr1 receptor [7]. In addition, iron deficiency may be another stimulus for FGF23 production through hypoxia-inducible factor-1a (HIF-1a) [8]. Furthermore, recent research in a mouse model of CKD demonstrated that interleukin 1β (IL-1β), a proinflammatory cytokine, acts as a key factor in the upregulation of FGF23 in the early stages of the disease. Administration of a neutralizing antibody against IL-1β effectively inhibited FGF23 expression [9]. Figure 1 summarizes the stimulants of FGF23.

FGF23, renin-angiotensin-aldosterone system, and hypertension

Chronic activation of the renin-angiotensin-aldosterone system (RAAS) has been shown to have hypertensive, prohypertrophic, and profibrotic effects on cardiac cells. Renin converts angiotensinogen to angiotensin I, and angiotensin-converting enzyme (ACE) metabolizes angiotensin I to angiotensin II, which causes high blood pressure and leads to myocardial hypertrophy and fibrosis. In another axis of the RAAS, ACE II degrades angiotensin I and II into angiotensin 1-9 and 1-7, respectively, which act as vasodilators and hypotensive agents. High levels of FGF23 have been shown to directly stimulate the RAAS by inhibiting ACE II, promoting vasoconstriction [10]. In addition, FGF23 can induce the expression of angiotensinogen, and in turn, angiotensin II stimulates the production of FGF23. Angiotensin II can regulate the bone expression of FGF23, thereby increasing its circulating levels with an added effect on hypertension. Likewise, aldosterone stimulates the bone expression of FGF23 [11].

Moreover, 1,25-dihydroxyvitamin D deficiency mediated by FGF23 activates the RAAS. This deficiency in mice promotes renal renin expression and the subsequent production of the vasoconstrictor angiotensin II, leading to the development of hypertension [12]. In a cohort study, higher levels of FGF23 were shown to be associated with increased systolic and diastolic blood pressure during a 10-year follow-up, with 35.2% of participants developing hypertension [13]. In addition, FGF23 may contribute to sodium management in the kidney independently of the RAAS [14]. In mouse models, it was found that FGF23 infusion increased tubular sodium reabsorption. Mice lacking the FGF23 gene excreted more sodium in their urine, while mice with high FGF23 levels had increased plasma volume, hypertension, and cardiac hypertrophy [15]. The study suggested that FGF23 stimulates sodium reabsorption and volume expansion through the sodium-chloride (Na-Cl) cotransporter [15].

FGF23 and chronic kidney disease (CKD)

Several studies have shown that FGF23 levels increase as the glomerular filtration rate decreases in both children and adults with CKD as a result of the decrease in functional nephrons, the excreted fraction of phosphate decreases, and this is detected by specific extracellular sensors, such as the PiT1/PiT2 cotransporters in osteocytes, FGFR1c, and the calcium-sensitive receptor (CaSR). These mechanisms contribute to the early stimulation of FGF23 secretion, which precedes the development of overt hyperphosphatemia, hypocalcemia, and secondary hyperparathyroidism [16-18]. From the early stages of CKD, a decrease in the levels of Klotho has also been observed, a protein discovered in 1997 by Makoto Kuro with anti-aging, anti-oxidative stress, and anti-fibrotic activities, among others, and whose main source of production is the proximal tubules of the nephrons [19]. The elevation of FGF23 appears to precede the decrease in Klotho, which is why both alterations are currently considered to be among the first to occur in CKD and responsible for the high mortality rate in this group of patients [20].

Elevated levels of FGF23 are associated with faster progression to end-stage renal disease. This has been explained after observing that FGF23 forces residual nephrons to increase the excreted fraction of phosphate, which generates tubulointerstitial inflammation, thus promoting fibrosis and the consequent progression of CKD [21]. Furthermore, in CKD, increased FGF23 suppresses the synthesis of 1,25-dihydroxyvitamin D by inhibiting 1α-hydroxylase, resulting in vitamin D deficiency. This reduces the absorption of phosphate from the diet in the intestine, avoiding further accumulation of phosphate in the body. However, this vitamin D deficiency in turn has many deleterious effects on the health of patients with CKD [22]. Vitamin D has been shown to have receptors and effects in various tissues of the body, being responsible for the pre-transcriptional control of multiple proteins. In this review, we will mention two. In the gene that codes for PTH production in parathyroid cells, there is a vitamin D response element that, when it detects a deficiency (<20 ng/mL), stimulates the transcription of that gene, thereby increasing the production of this hormone, leading to secondary hyperparathyroidism, an early alteration of CKD with a significant impact on bone and cardiovascular tissue [23].

**Figure 2:**
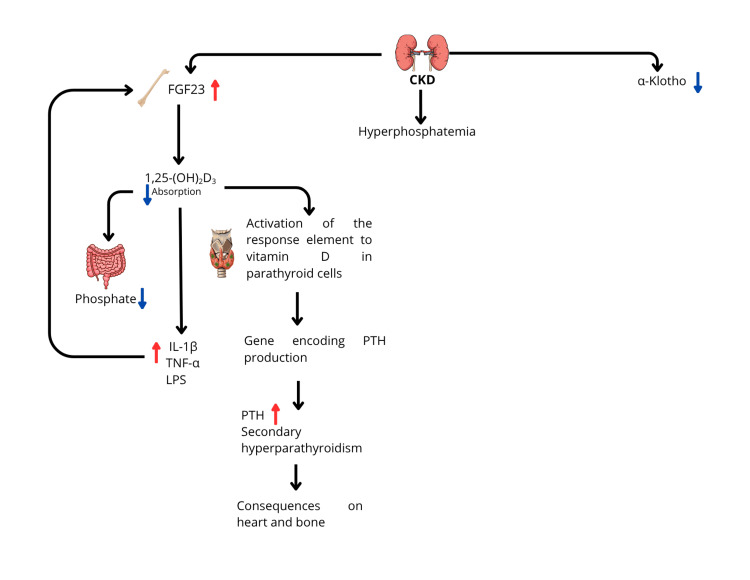
Effect of FGF23 on chronic kidney disease (CKD) CKD: Chronic kidney disease, FGF23: Fibroblast growth factor 23, 1,25-(OH)₂D₃: 1,25-dihydroxyvitamin D₃, IL-1β: Interleukin-1β, TNF-α: Tumor Necrosis Factor-α, LPS: Lipopolysaccharide, PTH: Parathyroid hormone. Image created by the authors.

Furthermore, vitamin D has an inhibitory effect on the nuclear factor kappa-light-chain enhancer of activated B cells (NF-kB), which represents a family of transcription factors that regulate genes involved in the immune response and inflammation, such that in states of vitamin D deficiency, an increase in inflammation markers has been demonstrated [24]. It has also been shown that some proinflammatory markers, such as IL-1β, tumor necrosis factor alpha (TNF-α), and lipopolysaccharide (LPS), stimulate the production of FGF23, which in turn perpetuates the progression of CKD, creating a vicious cycle [21]. Figure 2 summarizes the effect of FGF23 on CKD.

FGF23 and atherosclerosis

An association has been found between elevated levels of FGF23 and atherosclerosis, as well as calcification and thickening of the intima and media of the carotid artery. In a study of 764 Chinese men with normal renal function, elevated serum FGF23 levels were found to be an independent and positive factor associated with carotid intima-media thickness [25]. In addition, FGF23 and phosphate were associated with greater maximum carotid plaque height. In women, FGF23 was correlated with greater calcification in plaque lesions, while in men it was associated with the fatty components of plaque [26]. A cross-sectional study involving 403 subjects found that elevated FGF23 levels and the FGF23/Klotho ratio were positively associated with carotid intima-media thickness and carotid atherosclerosis in patients with type 2 diabetes mellitus [27]. In the CORDIOPREV study, individuals in the third and fourth quartiles of FGF23 exhibited a 1.9- and 2.1-fold higher risk of experiencing an atherosclerotic event compared with those in the lowest quartile; however, there is no significant causal link [28]. The mechanisms of association between FGF23 and subclinical atherosclerosis are still unclear [29].

FGF23 and endothelial dysfunction

Elevated FGF23 levels are associated with endothelial dysfunction and small vessel disease. One study investigated the direct effects of exogenous FGF23 on vascular function using the mouse aorta and found that FGF23 inhibits endothelium-dependent relaxation. This FGF23-mediated impairment of endothelial function was attributed to reduced nitric oxide availability [30]. Another study demonstrated that FGF23 stimulates the production of reactive oxygen species (ROS) in endothelial cells, inducing oxidative stress; however, the ROS balance is modulated by the presence of Klotho. In CKD, where FGF23 is elevated and Klotho is reduced, oxidative stress in endothelial cells results from excessive ROS generated by FGF23, leading to endothelial dysfunction [31].

FGF23 and left ventricular hypertrophy

Elevated FGF23 causes various structural alterations in the heart. One of the first to be described was left ventricular hypertrophy (LVH), observed in the hearts of experimental rats that suffered hypertrophy after intramyocardial injection of a solution containing FGF23. The same finding was observed when the injection was intravenous [32, 33]. Today, we know that the signaling pathway that mediates left ventricular hypertrophy in patients with CKD is carried out through the activation of the FGF23 type 4 receptor, identified as a specific FGFR isoform in the heart. When FGFR4 is activated, it recruits and phosphorylates phospholipase C (PLC), subsequently activating the calcineurin/NFAT signaling cascade, a potent inducer of ventricular remodeling in response to various pathological stimuli. This receptor conditions the prohypertrophic action of FGF23 even in the absence of Klotho. Incidentally, signaling in the absence of Klotho occurs mainly when FGF23 concentrations are extremely high, which is characteristic of patients with CKD [34]. A second prohypertrophic pathway of FGF23 could be through the direct regulation of the sodium chloride channel in the renal tubules mediated by FGF23, becoming a sodium-conserving hormone [35]. There is currently sufficient evidence of the effect of elevated levels of FGF23 and LVH [36].

FGF23 and heart failure

Experimental evidence has indicated that the FGF23/Klotho axis is involved in left ventricular remodeling and sodium reabsorption [37]. Left ventricular hypertrophy is prevalent in patients with CKD and leads to increased diastolic filling velocity and pressure, a pathogenic mediator of heart failure (HF) with preserved ejection fraction [38]. One study mentions the relationship between FGF23 and B-type atrial natriuretic peptide (BNP), which are associated with an increase depending on the degree of right ventricular dysfunction, only being relevant in patients who are in a severe state of HF with reduced ejection fraction, regardless of whether there is congestion in the systemic circulation or a subjective perception of congestion. The relationship between FGF23 levels and the degree of right ventricular dysfunction is likely to be non-linear, as there is currently no specific biomarker for right ventricular dysfunction [39]. Elevated FGF23 is associated with a significantly increased risk of incident HF in hypertensive patient populations [40].

FGF23 and atrial fibrillation

Elevated plasma levels of FGF23 have been associated with a higher incidence of atrial fibrillation and left ventricular dysfunction [41]. The induction of prohypertrophic genes in cardiomyocytes mediated by elevated serum FGF23 levels leads to hypertrophy of the left chambers of the heart. Therefore, this causes diastolic dysfunction and atrial dilation, which induces atrial fibrillation [42]. In patients with CKD, Klotho deficiency may increase the cardiac toxicity of FGF23 and promote cardiomyocyte aging, thereby favoring the incidence of atrial fibrillation. As a result of atrial fibrillation, the atrium undergoes fibrosis due to abnormal metabolism of type I and type III collagen and disorders in the alignment of these same collagens, which further induce the onset of atrial fibrillation [43].

FGF23 and myocardial infarction

The role played by FGF23 in myocardial infarction is still unclear, although it is known that it is produced by bones. Immediately after a heart attack, cardiac fibroblasts are responsible for generating FGF23 during the inflammatory phase through the stimulation of proinflammatory cytokines such as IL-1β and TNF-α. Two to three weeks after the infarction, the proliferative phase begins, in which transforming growth factor beta (TGF-β) inhibits the expression of FGF23. This suggests that FGF23 could play a natural therapeutic role after myocardial infarction [44]. In a study investigating the association between 92 inflammatory markers and mortality within the first 28 days after ST-segment elevation myocardial infarction (STEMI), the significantly associated biomarkers were IL-6, IL-10, IL-8, MCP-1 (mainly inflammatory markers), FGF-21, ST1A1, 4E-BP1, and CST5 (classified by OLINK (Olink Proteomics, a high-sensitivity proteomics platform based on Proximity Extension Assay (PEA) technology) as cardiovascular markers) and FGF23 [45]. In another study of patients with acute myocardial infarction admitted to Augsburg University Hospital, the results suggest that plasma FGF23 concentrations rise after this event and do not support the idea that elevated FGF23 concentrations are a risk factor for acute myocardial infarction [46].

FGF23 and myocardial fibrosis

In addition to bone production, FGF23 has been shown to be produced in cardiac tissue by local fibroblasts. Its functions include increasing fibroblast migration and the expression of fibronectin and type I collagen [47]. Stress and cardiac injury induce angiotensin II and aldosterone to promote the differentiation of cardiac fibroblasts into activated myofibroblasts. The latter proliferate and migrate within cardiac tissue to induce collagen synthesis and extracellular matrix remodeling, ultimately leading to fibrosis and cardiac dysfunction. It is still unclear whether FGF23 conditions cardiac fibrosis through the calcineurin/NFAT pathway or by activation of the RAAS [48]. Ischemic heart disease and cardiac muscle fibrosis are considered two of the main causes leading to HF and arrhythmias [49].

FGF23 and mortality

One of the first to report the association between FGF23 levels and high mortality was Orlando-Gutiérrez, who observed among patients with stage 5 CKD on dialysis grouped into four quartiles according to their FGF23 levels that the higher the FGF23 level, the higher the mortality rate [50], this same association was reported in the chronic renal insufficiency cohort (CRIC) and HOST studies conducted in patients who had some degree of CKD but had not yet started dialysis [51, 52]. The same results were found by other authors in transplant patients and, more surprisingly, in the general population without kidney damage [53, 54]. All these studies demonstrate the deleterious effect of FGF23 on the cardiovascular system and explain why this molecule is now considered an excellent biomarker of cardiovascular risk.

FGF23 treatment strategies

The recognition that elevated FGF23 and decreased Klotho are the first two biochemical alterations observed in the earliest stages of CKD (stages 1 and 2) has led us to the need to implement strategies to control these molecules in order to minimize their deleterious effects [55]. In the early stages of CKD, the best control strategy is to educate patients on low-phosphate diets, with a primary emphasis on avoiding the consumption of inorganic phosphorus contained in food additives in preserved foods (canned and bottled), as these have a bioavailability of 90 to 100%, meaning they are very easily absorbed by the intestine and enter the bloodstream [56]. In more advanced stages of CKD, pharmacological strategies to lower FGF23 levels include non-calcium phosphate binders such as sevelamer and nicotinamide (vitamin B3), calcimimetics such as cinacalcet, and non-pharmacological strategies such as parathyroidectomy in patients with severe secondary hyperparathyroidism (PTH levels greater than 1000 pg/mL without remission after pharmacological treatment) and dialysis in its various forms [57].

Burosumab is a recombinant human monoclonal antibody (IgG1) that binds to FGF23 and inhibits its activity, but to date, it is only indicated for XLH-linked hypophosphatemia; it is not indicated for CKD due to the risk of severe hyperphosphatemia that patients may experience [58]. In the future, the discovery of a drug that can selectively block FGF-23 receptors, especially type 4, which is expressed in cardiac tissue and is primarily responsible for all FGF-23-induced alterations at this level, could be a very valuable therapeutic resource in clinical practice.

## Conclusions

FGF23 has emerged as a critical regulator of phosphate metabolism and has significant implications for cardiovascular health. Its elevation, common in diseases such as CKD, is associated with an increased risk of cardiovascular events and death, manifesting as left ventricular hypertrophy, HF, endothelial dysfunction, ischemic heart disease, cardiac fibrosis, and atrial fibrillation. Multiple mechanisms through which FGF23 can affect the cardiovascular system have been recognized, such as its interaction with the RAAS and the induction of oxidative stress. However, further research is needed to determine which of these established interventions is definitively causal.
